# Dairy farmers’ considerations for antimicrobial treatment of clinical mastitis in British Columbia and Alberta, Canada

**DOI:** 10.3389/fvets.2024.1417958

**Published:** 2024-08-08

**Authors:** Ellen de Jong, Inge van der Velden, Anne-Marieke C. Smid, Jennifer A. Ida, Kristen K. Reyher, David F. Kelton, Herman W. Barkema

**Affiliations:** ^1^Faculty of Veterinary Medicine, University of Calgary, Calgary, AB, Canada; ^2^Faculty of Veterinary Medicine, Utrecht University, Utrecht, Netherlands; ^3^Department of Population Medicine and Diagnostic Sciences, Cornell University, Ithaca, NY, United States; ^4^University of Bristol Veterinary School, Bristol, United Kingdom; ^5^Department of Population Medicine, Ontario Veterinary College, University of Guelph, Guelph, ON, Canada

**Keywords:** clinical mastitis, decision-making, antimicrobial use, treatment choice, farmer attitudes

## Abstract

**Introduction:**

Clinical mastitis (CM) treatment decision-making is a multifaceted process that remains relatively understudied, despite CM being one of the most prevalent diseases on dairy farms worldwide, contributing greatly to the use of antimicrobials in the dairy industry. This study aimed to gain insights into decision-making mechanisms employed by dairy farmers in British Columbia and Alberta, Canada, when dealing with CM.

**Methods:**

Interviews were held with 15 dairy farmers in the Canadian provinces of British Columbia and Alberta and analyzed using reflexive thematic analysis to develop both the decision-pathway and overarching themes influencing the CM decisions by farmers in this region.

**Results and discussion:**

The analysis generated a decision-making process that begins with identification and classification of CM, guided by visual characteristics of milk and the udder, available milk production and quality data, presence of systemic signs, and additional diagnostics. Subsequently, CM cases are assessed based on the likelihood of cure, value of the cow, and herd goals to decide whether antimicrobial treatment is desired. Next, a treatment choice is made by evaluating severity and urgency of the case, availability of drugs and timing of the case. Finally, definition of treatment success and progression over time following the treatment decision guides the termination of treatment. Three overarching themes were generated that shape the decision-making process: ‘Personal attributes’, including personal approach and experiential knowledge; ‘Inter-actor dynamics’, such as shared decision-making and dynamics among producers, veterinarians, and milkers; and ‘Moving beyond protocols’, which highlights the dynamic nature of mastitis decision-making. These insights have the potential to inform the development of effective interventions to improve CM antimicrobial use that align with the reality of farming operations within Western Canada, and potentially beyond.

## 1 Introduction

Mastitis is an inflammation of the udder, marked by various physical and chemical changes in the milk and the udder tissue (1). Visual confirmation of changes to the milk classifies clinical mastitis (CM), with more moderate cases also showing inflammatory signs in the affected quarter(s), and severe cases including signs of systemic illness (2). While yeast, algae, external particles, and udder trauma can cause mastitis, CM is predominantly caused by bacterial pathogens. Therefore, intramammary antimicrobial administration is the recommended and widely adapted treatment for CM, often accompanied by systemic antimicrobials and pain medication in severe cases (3).

Mastitis is one of the most prevalent diseases on dairy farms world-wide (4), with an average CM incidence in Canada of 19 cases per 100 cow-year (5). With treatment rates averaging between 90 and 100% (6–8), CM treatments contribute greatly to the total amount of antimicrobials used on dairy farms. When coupled with dry cow therapy, intramammary antimicrobial use accounted for 66% of total antimicrobial use on Canadian dairy farms during 2019 and 2020 (9). While direct implications of mastitis to the farm owner primarily include economic losses stemming from preventative measures, culling, reduced milk yield and milk discard—estimated at CA$744 per CM case (5)—implications of mastitis-related antimicrobial use extend to broader concerns surrounding antimicrobial resistance (10), which necessitates responsible use of antimicrobials on dairy farms. In addition, due to the frequent nature of mastitis, in many countries including Canada, farmers are allowed to administer antimicrobial intramammary treatments following protocols and antimicrobials prescribed by their herd veterinarian, adding depth to their role as farmer.

In the context of mastitis management, many Canadian dairy farmers have embraced practices aimed at improving udder health within their herd, such as proper milking procedures and vaccines, although the latter are still not widely adopted (5, 11). The practice of analyzing CM milk samples to guide antimicrobial treatment decisions has existed for some time, their importance to reducing antimicrobial use further highlighted in recent efforts (12). However, the adoption of analyzing CM samples is less than optimal, as evidenced by a survey among Canadian farmers (8). To ensure effective adoption of such interventions and to enhance antimicrobial stewardship among dairy farmers, a comprehensive understanding of on-farm CM treatments decision behavior remains critical (12, 13) and is often overlooked when implementing or suggesting interventions, leading to a lower-than-expected compliance (14).

The decision-making process for treating CM with antimicrobials is multifaceted and has a complex decision structure. Vaarst et al. (15) described in the early 2000s for Danish dairy farmers, that their decision-making included weighing various information sources including SCC, CM case history, lactation stage, reproduction status, value of the cow, availability of replacement heifers, bulk tank SCC, and availability of alternative treatments. Recent survey results highlighted the differences in priority of these decision-factors among Canadian farmers (8). Although these results suggest that these priorities shape the decision-making process of individual farmers, the quantitative study design did not allow for an exploration of underlying causes. This is particularly of interest as research into general antimicrobial use has underlined that intrinsic and extrinsic factors impact antimicrobial use decisions. These factors include attempts to increase the chance of cure by treating as quickly as possible, motivation to improve animal welfare, perceived efficacy of chosen treatments, and external referents such as other farmers and the herd veterinarian (16–18). Although it is unsure how these factors play a role in CM decision-making.

To address the lack of a comprehensive and nuanced understanding of CM-related antimicrobial treatment decisions among dairy farmers, given the modern landscape where numerous tools and techniques are available to the farmer to enhance udder health and refine antimicrobial treatment choices, this study used a qualitative approach to investigate the core of dairy farmers’ practices and decision-making mechanisms around CM treatment decisions. As opposed to quantitative research, qualitative research can facilitate in-depth exploration with farmers, thereby allowing participants to share their experiences, priorities and thought processes. Interviews also offer a personalized and contextual understanding. As such, the qualitative approach of this study will be able to capture insights in CM decision-making that quantitative methods might overlook.

## 2 Materials and methods

This study was reviewed and approved by the University of Calgary Conjoint Faculties Research Ethics Board (# REB21-0699). This report was written according to the Consolidated Criteria for Reporting Qualitative Research (COREQ) framework (19).

### 2.1 Positionality statement

The lead author (EdJ) is female, 29 years old, and holds a PhD in Veterinary Medical Science from the University of Calgary. Her PhD research focused on mastitis-related antimicrobial use on dairy farms, a topic on which she and her colleagues have published several manuscripts. EDJ’s knowledge of the dairy industry came from research projects conducted in the Netherlands and Canada as part of her BSc and MSc degrees in Animal Sciences at Wageningen University in the Netherlands, and from her work in her PhD. EdJ comes from a family of dairy farmers, and though her grandparents were the last ones to own a farm, many relatives still work in the agri-business. EdJ lived in the Netherlands until the age of 24 years, and thus shares a cultural background with many of the participants as Dutch ancestry is common among Western Canadian dairy farmers. EdJ is a dairy product consumer and a left libertarian according to the economic/social political compass. Since EdJ’s research has been largely quantitative in nature, she familiarized herself with qualitative research methods and analysis through auditing courses, extensive reading, and collaboration with colleagues with experience using qualitative methods. All other authors work or study in dairy veterinary science and have a range of training in, and experience with, qualitative research methodologies.

### 2.2 Data collection

Data were collected through semi-structured interviews between June 2022 and August 2023. Participants in the Canadian provinces of Alberta and British Colombia were recruited through existing connections of the researchers and through extension events. As such, participants were aware of the study interests of the researchers. Potential participants were shortlisted based on their herd size, milking type, and location to ensure a variety in farm management practices.

A telephone script was used for recruitment by phone. During the recruitment, special attention was paid to scheduling the interview with those responsible for most CM treatment decisions. Consent was sought either on paper prior to the interview or orally, if the participant was unable to sign the paper consent form ahead of the interview. A short online questionnaire, using the software platform Qualtrics (Seattle, WA, United States), was sent ahead of the interview to acquire information regarding milking system, number of milking cows and production parameters for each farm (https://data.mendeley.com/datasets/hv9h7k499b/1; 8).

Interviews were conducted using Zoom (Zoom Video Communications, San Jose, CA, United States) or in-person upon the producers request if the location of the farm was within 200 km of the University of Calgary. One interview was conducted by phone due to lack of an internet connection on the farm. In-person and phone interviews were voice-recorded using 2 recording devices (Philips VoiceTracer DVT2050). Zoom-interviews were video- and voice-recorded using both the recording device as well as the Zoom recording function. All interviews were conducted by EdJ, except for 5 interviews which were conducted by EdJ and IvdV together.

Interviews were guided by a semi-structured interview script and consisted of 3 parts (https://data.mendeley.com/datasets/hv9h7k499b/1; 8). First, questions were asked to build rapport and gain an understanding of the farmer’s background and the farm itself, including personnel situation and the farm’s strengths and challenges. Throughout this text, ‘farmer’ refers to an individual who has financial ownership and investment in the farm. In the case of some participants, this person may also participate as a milker, train employees on recommended milking procedures, and, in collaboration with the herd veterinarian, may assist in the development and implementation of mastitis diagnosis and treatment protocols. This individual may also be responsible for relaying that information to other on-farm employees who will implement established protocols. All participants were in a position of decision-making autonomy either solely or in collaboration with another farm owner. Secondly, participants were asked to describe their most recent CM cases, using their own definition of CM. Follow-up questions covered case identification, information sources consulted, communication between farm personnel and veterinarians, treatments considered (both antimicrobial and non-antimicrobial, including anti-inflammatory), and treatment expectations. Various CM cases were discussed with each participant, until both the interviewer and the interviewed farmer were satisfied that the full range of scenarios on the farm had been discussed. Lastly, questions were asked regarding on-farm antimicrobial stewardship, which will be analyzed and published separately. The interview guide was not pretested, but the researchers revised and adapted the guide in an iterative fashion at several instances during the data collection process to further explore generated themes. The interviewers were free to rephrase questions and use probing questions throughout the interview.

Duration of interviews averaged 55 min ranging from 35 to 79 min. The interviews were transcribed verbatim with the help of the automated transcription program Otter.ai (Los Altos, CA, United States) and checked for accuracy. The first 5 interviews transcripts were checked by both IvdV and EdJ, the other 10 transcripts were checked by EdJ. Participants received an anonymous participant ID and are referred to in the paper as P#.

### 2.3 Analysis

The CM decision-making process can be classified as multifaceted and complex in nature (20), which warrants considering them within a broad context that goes beyond traditional hierarchical decision research (21, 22). Reflexive thematic analysis was therefore chosen for this research, as its reflective approach allows for use of the critical realist paradigm compared to the constructivist paradigm of the classical decision research concept (23–25).

All interviews were analyzed by the principal researcher (EdJ). After familiarization with the data, segments of text that captured meaningful information related to the farmers’ CM treatment decisions were paraphrased into codes. The inductively generated codes were recorded in the online diagramming tool draw.io (JGraph Ltd) and collated to generate the different facets of CM treatment decisions, as well as to simultaneously generate themes that ‘overarch’ the core concepts of the CM treatment decision pathway. Codes and themes were refined and revised by re-reading transcripts and identifying quotes to provide context. This iterative process, in combination with extensive discussions between EdJ and A-MS regarding data interpretation, ensured rigorous and insightful representation of the data. After the analysis was finalized, all participants were offered the opportunity to validate the draft manuscript to ensure that their views were accurately captured (26).

An assessment of ‘information power’ was used to identify whether enough interviews were conducted to obtain a good understanding of farmer decision making regarding clinical mastitis treatment in Western Canada. Information power is preferred over ‘data saturation’ in reflexive thematic analysis (25), because it aligns with the understanding that meaning is generated through interpretation rather than being discovered, making the determination of sufficient data inherently subjective and context-dependent (27). Information power suggests that the more relevant information the sample holds for the actual study, the fewer participants are needed (28). Information power depends on the width of the study aims, sample density, the use of an established theory, or not, quality of dialog, and analysis strategy (28), and was assessed continuously throughout the iterative process of data collection and analysis to determine the sample size.

## 3 Results

Fifteen interviews were conducted with 17 dairy farmers in the Canadian provinces of Alberta (*n* = 11) and British Columbia (*n* = 6). In total, 24 farmers were approached, of which 4 could not participate due a lack of time, 3 were unable to respond to scheduling requests, 1 did not feel the interest of the study aligned with their farm goals, and 1 was no longer in operation. Participants were either farm owners or herd managers, each actively involved in CM diagnosis, treatment decisions and udder health management. The farms were a mixture of those milking in a parlor (n = 8) and with automated milking systems (AMS) (*n* = 7). One farm produced grass-fed milk; the other 14 farms operated conventionally, meaning they were not required to adhere to specific production labels such as organic or grass fed. As organic dairy farms have to adhere to more strict regulations regarding AMU (29) which can affect decision-making mechanisms on treatment of CM, organic dairy farmers were excluded from this study. One farm was part of a Hutterite colony. Hutterites, a German speaking community, have a distinct communal structure and are accountable for a substantial portion of dairy production in Alberta (30). Almost all farms were staffed by a combination of family members and external employees, with the larger parlor farms employing additional individuals to aid with milking. Most interviews were conducted with 1 interviewee, whereas during 2 interviews 2 interviewees were present. In total, 15 farmers were male and 2 were female. Production characteristics of the dairy farms are described in Table 1; study sample averages are in accordance with provincial averages.

**Table 1:** Milk production characteristics of the study farms (*n* = 15) associated with the participants interviewed (*n* = 17) between June 2022 and July 2023.

	Min	Max	Median	Provincial means^1^	
				BC	AB
No. lactating cows	93	385	137	190	164
Current average daily milk yield (kg)	32.1	42.0	37.0	31.7	32.9
Current milk fat %	3.7	4.7	4.3	4.4	4.3
Current milk protein %	3.1	3.5	3.3	3.3	3.3
Average BTSCC^2^ last month (cells/mL)	70,840	251,000	154,000	145,951	162,298

^1^On January 2023, extracted from the Canadian Dairy Information Centre on November 26, 2023 (https://agriculture.canada.ca/en/sector/animal-industry/canadian-dairy-information-centre).

^2^Bulk tank somatic cell count.

### 3.1 Clinical mastitis decision pathway

**Figure 1:**
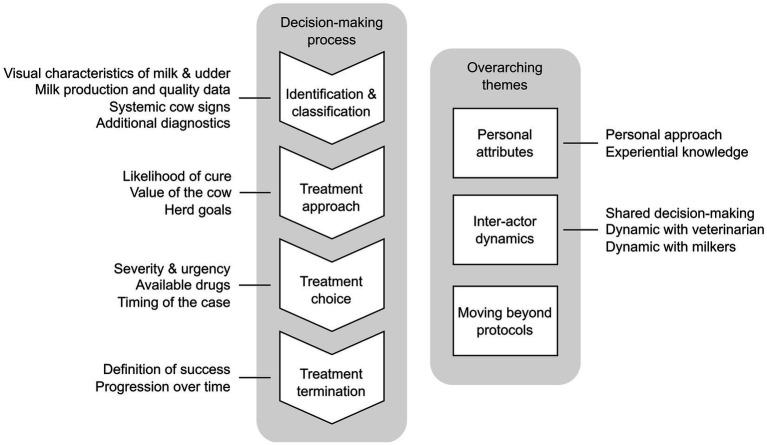
Sequence of considerations for antimicrobial treatment of clinical mastitis cases and overarching themes, based on interviews (*n* = 15) with Canadian dairy farmers (*n* = 17) in Alberta and British Colombia between June 2022 and July 2023.

Upon thematic analysis of the transcripts, a decision pathway (Figure 1) was developed that all farmers followed regarding their CM antimicrobial treatment decisions. This pathway included various considerations at each step. Identification and classification of CM cases formed the start of this pathway (which differed depending on milking system), followed by an evaluation of likelihood of cure, perceived value of the cow, and herd goals to arrive at a treatment approach. Next, treatment options were considered based on severity, drug availability, clinical signs, and timing of the case. The decision pathway ended with an assessment of treatment expectations and progression to determine treatment termination.

#### 3.1.1 Identification and classification

All participants described direct visual confirmation of clinical signs to be most important in identifying CM, which includes presence of flakes, discoloration, and milk consistency, as well as hardness and temperature of the udder quarters. One farmer described this as: “Usually you see any abnormality like the color of the milk, or any kind of swelling of the quarter or chunks or watery milk or anything like that” (P4). These observations often took place during the fore-stripping process in the parlor.

Some farmers described their high proficiency to identify CM cases by relying on subtle indicators such as differences in udder shape. One farmer described: “You use a teat dip cup. You touch the udder with the top. So, you have another option that you think like hey, this is maybe not right” (P6).

Next, to visual identification in the parlor, participants also used milk production and milk quality data to shortlist cows with a high likelihood of CM, after which identified cows were subjected to visual examination. Specific indicators include changes in milk production, SCC data obtained from DHI records or AMS, conductivity, instances of milk filters clogging due to the presence of clots or cows milked with AMS who had incomplete milkings: “I look at my cell count list when I do DHI, and I’ll see if there’s anybody high that’s surprising” (P4), and “As soon as one cow has something that will stick on the milk filter, it is noticeable. So really, that’s the first line” (P13). Indicators were used alone or in combination. On farms with AMS, missed visits to the AMS were also viewed as an indicator for udder health problems: “I’d say my biggest ones are they are not going to the robot and they are down in milk” (P17).

The selection of the milk production and quality indicators was guided by data availability. However, these data were not prioritized and valued similarly by the interviewed farmers. For example, for one farmer low milk production was the starting point of CM identification: “When she comes into the parlor and there’s little milk yield, we’ll go back to the data” (P15). After having identified cows with a high likelihood of CM, the California Mastitis Test (CMT) was typically used to pin-point the inflamed quarter. A farmer detailed this process, explaining: “We’ll flag them in the parlor so that we see them. And then we’ll do a California Mastitis Test on them” (P16).

The third route to identify CM included an overall assessment of the cow’s wellbeing, including behavioral pain indicators such as kicking during milking, reduced activity levels, decreased rumination and other systemic inflammation signs such as fever. This was described by one producer as: “If we were to visually see that the cow is dropping in milk, or if she’s looking skinny—we also have the Heatime system [real-time monitoring through ear or neck tag; Heatime® Pro+, Allflex, Saint-Hyacinthe, QC, Canada], so the rumination and the activity. If we start seeing them going down that way, we would intervene” (P5).

More specifically, for severe CM cases, farmers described the presence of a hard quarter, watery milk, a severe decline in milk production, a sore udder, or behavioral pain indicators such as kicking: “She’s downhill for real. Milk clear, watery looking, and swollen udder, fever” (P16). A rapid onset of these clinical signs was also mentioned as an indicator for severity: “… speed of onset and severity of symptoms. If the whole quarter is packed right full of junk right away then I’m not gonna put out, I’ll treat her right away” (P4). Severe cases that included the combination of a hard quarter and watery milk were referred to by many farms as “*coliform”* mastitis, or “*E. coli”* despite the absence of diagnostic culturing to confirm causal pathogen: “And then we have coliform which comes out as orange [milk] and the udder is hard, rock hard” (P17).

Definition of chronic cases varied among the interviewed farmers, but commonly mentioned were cows with persistent high SCC and those with frequent recurrence of CM, as detailed by the farmers: “They have flakes, and they do not go away. Every milking, they come in and you strip them out and you’ll mint the udder and for weeks” (P1), and “… anything that regularly has a cell count over 250–300,000” (P4).

The interplay of these three identification pathways: (1) direct visual confirmation of clinical signs and milk characteristics, (2) cows with a high likelihood of CM based on specific indicators, and (3) overall assessment of the cow’s wellbeing was intricate and farm-specific and was further influenced by the milking set-up (AMS vs. parlor). For example, on AMS farms, identification through data often preceded visual checks. One of the interviewed farmers milking with an AMS explained: “I track our mastitis through conductivity per quarter via the robots, and from there we paddle test with CMT, and make our assessment from overall look of the cow, the look of the milk, the volume of the milk and how well the cow is eating” (P14). In contrast, on parlor farms, visual confirmation overall preceded analyzing data, as other farmers who milk in a parlor explained: “Then [after the milker calls with a case of mastitis] I pop onto the computer and check the history, whether she was down in previous milkings or not” (P1). All farmers underscored the significance of visual confirmation of clinical signs before treating a cow: “I would want to be seeing a drop in milk, I want to be seeing changing [milk] color, CMT paddle tests, all that before treatment goes into place” (P14).

Farmers also highlighted the significance of other subtle differences in identification of CM in AMS vs. parlor systems. AMS are less able to detect flakes in the milk, which can change the perception of CM by the farmer. As one farmer stated: “When we were pre-stripping in the parlor and she had pieces, we would treat them. But now you have got the robots. And you do not see all the pieces, and the cows are fine. So, it has been a gradual progression here. Mastitis is always going to be there, but it does not all have to be treated” (P10). However, it was also mentioned that AMS allow for more frequent SCC reports, making an interpretation of high SCC values possible within the context of an extended period: “Now that we are every day seeing their somatic cell score, and you realize that what we would have thought with DHI as a clinical case of this cow, that 2 million, in robots, she might be 2 million for a few days, and then she’s down to nothing” (P5).

Some farmers adopted an analytic approach by regular culture of milk samples through their DHI laboratory or veterinary clinic. Although some farmers cultured each CM case, it was more common among the interviewed farmers to only submit samples of specific cows or cases for culturing to confirm the presence of pathogens in the udder or determine the causal agent of an abnormal cluster of CM cases: “To see whether they actually had bugs in the quarters, or if it was just inflammation causing flakes to show up” (P1). Testing fresh cows was common, as described by one farmer: “We test all the fresh cows for *Staph.* at day six and day eight” (P12). This method allowed for the identification of intramammary infections at the start of the lactation. Seldomly were diagnostic tests used to evaluate treatment efficacy: “It’s when we see issues with the treatment, like if it’s not working being effective, then we’ll culture to see if there’s a new bug, or if we should be using a different antibiotic” (P14).

Identifying other potential causes of clinical signs was also an essential aspect of making treatment decisions. Noting these served as an additional tool to identify cases that might not benefit from antimicrobial treatment. This ranged from improper milking settings to the presence of malformed teat ends: “Some older cows will potentially have a bad teat end, or something that’s just not perfect. It does not seem like it’s dangerous to them. It’s not milk you want to drink or ship, but they will recover on their own” (P13).

#### 3.1.2 Treatment approach

After CM cases were identified, a treatment approach was determined. Farmers described starting with an assessment of the potential efficacy of antimicrobial intervention as compared to non-antimicrobial approaches, followed by an evaluation of the value of the cow, herd goals and weighing of alternative treatment options. These aspects interacted with each other and were considered together to determine a treatment approach with or without antimicrobials.

To determine the likelihood of cure when administering antimicrobial treatment, the same indicators were often used as those used for identification and classification of CM cases. More specifically, where *changes* in milk production, SCC, and conductivity were used to identify CM cases, *farm-specific thresholds* were used by some to inform the likelihood of cure and presence of chronic infections: “like maybe a 50% drop in milk” (P1), and: “The vet considers a mastitis case anything over 400 [thousand]. To me, that’s not worth the headache or the treatment. Because give her some time, and she might drop back under 400 without treatment. Because somatic cell count numbers can be all over the place” (P8). If cases did not meet the threshold, antimicrobial treatment was often withheld.

The assessment of cure also included an evaluation of lactation stage. A more pro-active treatment approach was often taken with fresh cows experiencing CM, as the transition period is known to increase physiological stress. Farmers expressed worry about the occurrence of other health conditions, and progression to worse forms of mastitis in fresh cows if left untreated: “They’re under a big transition, so they just do not have any reserves. And I think that’s when it [mastitis] can go [get worse]. It can be more deadly. They just do not have the same fight, or the same immune response [as cows later in lactation]. Their body is worried about 100 other things” (P13).

For most cases, history of CM in the same quarter was evaluated, although thresholds and associated actions differed by farm. Where one farmer had a strict rule: “If it’s the third time, we never treat again” (P3), others also considered the timing of repeated cases: “I would say if they relapsed right away, we would have them on a beef truck. But if they relapse in a couple of weeks, we would retreat them” (P17). The reason for this threshold was explained as: “I usually find that the treatment does not work [in those instances]” (P17). Similarly, if cows were deemed ‘chronic’, it was likely that she will not receive treatment: “If she’s chronic, sometimes I do not treat her and [farm owner 2] will say ‘beef her’” (P17). By incorporating these different elements, farmers tailored their treatment strategies to individual CM cases.

For those farmers who used additional diagnostics by sending milk samples to a laboratory for bacteriological culture or cultured milk samples on farm, outcomes of these tests were almost never factored into decisions regarding the treatment approach. Instead, they informed general herd trends or allowed for identification of subclinical mastitis among fresh and chronic cows. However, farmers articulated a level of confidence gained from the test results: “This incubator did not change my protocol at all, because I should have treated her anyway. So, it was good. Yeah. But it is nice to know” (P1).

Non-severe CM treatment approaches were often tied closely to culling considerations. An assessment was made of the cow’s contribution to the herd’s overall value. As such, low producing cows, non-pregnant cows and cows with chronic mastitis were more likely to be put on the cull or do-not-breed lists instead of receiving treatment for non-severe CM. In contrast, pregnant or high-producing cows were less likely to be culled and were thus more likely to be considered for treatment: “If a cow was pregnant, and she has good genetics, and she’s at 200 days in milk, and she develops a chronic mastitis, we’ll just milk her and dump it in the ground for the rest of her lactation with the eye to either trying to clean up in the dry period, or the worst case scenario if we get one more calf out of her” (P3). Cows in their first lactation were also less likely to receive treatment and be culled instead: “If it’s a first lactation, forget it, she’s probably gone. We’re not going to mess around. First lactation heifers should have zero issues” (P17).

The farmer’s personal feelings toward specific cows also played a role. A cherished animal might receive more attentive care than her counterparts, potentially leading to antimicrobial treatment. One farmer explained: “If it’s secondary [case in lactation], and I really liked her [personally], then, you know, she has a good chance of being treated again” (P3).

Certain herd goals also came into play when selecting a treatment approach, including milk production incentives under the Canadian milk supply system and limitations imposed by provincial milk boards on the level of bulk tank SCC (BTSCC). In scenarios where mild CM cases did not adversely affect overall milk quality, farmers described opting to withhold antimicrobial treatment or stop treatment as soon as improvements were seen, to fulfill targets. One farmer described: “If I see enough improvement, then I do not continue for another [additional treatment] day because I do like to see milk in the tank” (P12), and another described: “Depending on how we are doing with our quota that month, they [cows positive for *Staph. aureus*] either get shipped, or if we need to, we keep milking them for a while” (P12).

#### 3.1.3 Treatment choice

After selecting a treatment approach, different treatment choices were considered by the interviewed farmers, depending on severity, drug availability, and other factors.

For severe CM cases, common practice among the farmers was to invoke an assertive and comprehensive treatment approach, which farmer P13 referred to as the ‘shotgun’ approach, where a combination of intramammary and systemic antimicrobials, alongside anti-inflammatories and pain medication were administered: “First, Cefa-Lak [cephapirin sodium] in the quarter. Then I will separate her out and if she has a fever of like, 102–103 [degrees Fahrenheit] and above, I’ll do Trimidox iv [trimethoprim-sulfadoxine]. And then, like, Banamine [flunixin meglumine] for pain” (P13). Such an aggressive therapeutic strategy was motivated by several critical factors: a sense of urgency to address the cow’s need promptly, to prevent further decline of the cow’s health, and to increase the cows’ survival chances, as underscored by the following statement: “before she goes septic, to get that IV antibiotics in her system. The main objective: save the cow” (P14). This also holds for CM cases that initially were classified as non-severe but showed a worsening of clinical signs in the following days.

For moderate and mild CM cases, intramammary antimicrobials were deemed sufficient by most of the interviewed farmers: “When a cow has mastitis, our main mode of treatment is Cefa-Lak [cephapirin sodium]” (P5), and cases were monitored for worsening of signs. Only a few farmers took a more aggressive approach for non-severe cases. For example, one farmer used a protocol where a 5-day course intramuscular antibiotic was provided for any case: “Everybody gets Metacam [meloxicam] because they usually come with a fever. Everybody gets intramammary. In that quarter, we use Cefa-Lak [cephapirin sodium]. And then everybody gets 5 days of Trimidox [trimethoprim-sulfadoxine] treatment” (P17).

Limited intramammary antimicrobial products were available to the interviewed farmers due to discontinuation of products and new regulations. For example, because of the discontinuation of the intramammary drug Special Formula 17,900 (a combination of penicillin G procaine, dihydrostreptomycin sulfate, novobiocin sodium, polymyxin B sulfate, hydrocortisone acetate, and hydrocortisone sodium succinate), alternatives were sought. One of the interviewed farmers described limited availability as the reason they used Cefa-Lak (cephapirin sodium), explaining: “It used to be 17,900; they do not make that anymore, right?” (P13). Another farmer co-administered a steroidal anti-inflammatory alongside intramammary antimicrobials: “In the Spectramast syringe [ceftiofur hydrochloride] we do add dexamethasone, just to get the inflammation out of the udder” (P1).

Participants were divided regarding co-administration of pain medication in cows with non-severe CM. Some farmers provided pain medication in the presence of certain clinical signs of non-severe CM cases: “If we see that their [cows with non-severe CM] quarter’s hard or they are looking a little sad, we will give them also a dose of Metacam [meloxicam]” (P5). Other farmers stated that they did not give pain medication to cows with non-severe CM: “A mild mastitis? Yeah, no, probably not. I would not give pain medication” (P7). These farmers conveyed the belief that non-severe CM does not appear to cause significant pain in cows, or that cows do not show and therefore experience pain in the same way as humans do. “They’re not painful, if they are still milking 50 to 70 percent of their expected milk volumes. They just have an infection, but it is not slowing them down; it is not disrupting their eating, it is not disrupting their production substantially” (P14). This perception influenced the decision-making process, resulting in a limited use of pain medication for non-severe CM cases.

In situations where cows developed CM nearing the weekend, farmers tended to adopt a more aggressive treatment approach, primarily driven by the constrained availability of veterinarians and limited options for culling during weekends. Farmers opted to mitigate the likelihood of further deterioration of the cows’ condition. This was illustrated by one interviewed farmer who said: “If it’s a Friday or a Thursday, I always treat them because we have to keep them for the weekend” (P17).

For non-severe CM cases that would not qualify for antimicrobial treatment according to protocols, udder mint cream was popular among some of the farmers, as well as essential oils (such as calendula). Cream and oils were also used for more severe cases in combination with antimicrobial treatments in attempts to offer relief to signs such as hard and swollen udders. Nevertheless, a sense of skepticism existed regarding the effectiveness of these alternatives, and their use often stemmed from the desire to take immediate action of non-severe CM cases without resorting to antimicrobials: “She would maybe have gotten better on her own. But maybe it feels good. You’re doing something” (P1). Only 2 farmers mentioned the use of oxytocin: “I’m under the belief that oxytocin will, right off the bat, potentially just let [the milk] down. I’m under the belief that if the cow can let it out, that’s, eh, less to fight” (P13).

#### 3.1.4 Treatment termination

Following the chosen treatment, each farmer described a process where CM cases were monitored until a set outcome was reached. These outcomes were influenced by the severity of the CM case at time of identification.

When asked about assessing the cure of non-severe CM cases, the interviewed farmers provided a comprehensive list of clinical signs and production characteristics as determining factors. These included the absence of flakes in the milk, lower SCC, resolution of udder tenderness, and restoration of milk production to normal levels. For example, one farmer shared: “You hope to see a cow with low somatic cell and milk production back to expected [levels] or higher” (P14).

For severe CM cases, treatment expectations were more temperate and included indicators such as an increase in milk production, absence of clinical and systemic signs, and survival of the cow. More specifically, farmers expressed pessimism regarding the recovery of cows from severe CM cases if substantial inflammatory signs were present at time of diagnosis: “If she does not respond within a few days, the outcome is not very good. Then she will probably be dead within five days” (P7).

Similarly, for chronic cases, treatment expectations were also tempered: “Chronic cases that kind of get it [CM], and then it goes away. And then they have a high somatic cell count for a while. Some of those ones do not always respond to treatment” (P16). Multiple farmers discussed the option of drying off quarters after unsuccessful treatment. For instance, one farmer shared: “If they are chronic, and treated them or they will not respond to treatment, we have dried off quarters in the past […] And then we’ll have a three-quarter cow who still milks good. Cheaper to keep her that way” (P16).

Practices diverged when the endpoint of antimicrobial treatment was discussed. Some farmers did not adhere to label instructions, discontinuing antimicrobial treatment after 2–3 days: “If they are still clinical, we will not just keep treating them because we should; maybe it’s already cleared up” (P16). Conversely, some farmers extended antimicrobial courses up to 5–7 days until all clinical signs had completely subsided. Some switched drugs during this period, although this was typically accompanied by a consultation with the veterinarian: “If the search [for a cure] gets longer than 4 or 5 days, I’ll start talking with my vet” (P4). Regardless, there was consensus that visible improvement of non-severe cases was expected within 3–4 days and cure was expected within a week when treatment was successful: “If we see a response, then we’d call that a success. I would assume within a week or so” (P5).

### 3.2 Overarching themes

In addition to the decision-pathway, three themes were generated from the interviews that ‘overarched’ every aspect of the CM treatment decision-pathway which can be described as ‘Personal attributes’, ‘Inter-actor dynamics’, and ‘Moving beyond protocols’.

#### 3.2.1 Personal attributes

##### 3.2.1.1 Personal approach

The influence of differences in approach among owners, herd managers, and milkers on dairies (where multiple people share the decision making) appeared to hold significant importance. Contrasting perspectives of the owner and the farm manager were evident during several interviews. In one interview, the owner leaned toward a more relaxed approach, preferring to observe the situation before acting, while the farm manager advocated for a proactive stance, leading to a more liberal use of antimicrobials for treating CM cases: “I [full-time farm hand] tend to treat them quicker. I am more like ‘Oh, she had flakes tonight, I’ll start treating her’. I’m quicker [to treat] where he [farm owner] will just wait and see what happens for 12 or 24 h” (P12). On this farm, it also translated into differing durations of intramammary antibiotic treatments: “I think that I [full-time farm hand] treat them longer than [farm owner] would have done too” (P12).

##### 3.2.1.2 Experiential knowledge

The evaluation of the factors in each step in the decision-making sequence (Figure 1) was often attributed to experience by the interviewed farmers. This included generational knowledge, where certain products have been used to treat CM previously, including by older generations of farmers: “The way our dad taught us is just basically give three treatments. Give Cefa-Lak [cephapirin sodium], let us say, for example, at 5 pm, and then the next day at 5 pm as well, and the next day at 5 pm. That’s the protocol at this point” (P9). Another farmer’s perspective exemplified this generational continuity regarding abstaining from treating certain cases: “For 16 years, my dad always had the idea: ‘If they get *E. coli*, you just got to let them be and they either fight it and win or they do not” (P5).

Experiential knowledge of outcomes of previous decisions was also important. Sometimes this pertained to previous CM cases in the same cow: “So, if we did not use Metacam [meloxicam] in the first case, or if we used Spectramast [ceftiofur hydrochloride], now we’ll use something else” (P3), and: “That one older cow, that [CM] will sometimes come in waves. We do not treat her and that’s just kind of the way I do it” (P13). Experiential knowledge also encompassed treatment outcomes of other cows with similar CM cases: “Sometimes you just think, it did not work for the last cow, is it going to work for this cow?” (P6). Farmers also linked the historical efficacy of certain treatment choices and products to treatment decisions: “I have been doing this for a little while. You can safely assume that miscellaneous case that recurs every three weeks, it’s probably a chronic Staph.” (P3), and: “Our vets have in the past said a saline solution IV helps, but we have tried, and we just give them time and put them in a straw pack and see what they do” (P5).

#### 3.2.2 Inter-actor dynamics

##### 3.2.2.1 Shared decision making

On non-AMS farms, milkers played a crucial role in identifying CM cases. Almost all interviewed farmers shared the on-farm labor with family members or external staff. The interviewed farmers detailed a close relationship between the milking staff and the farm owners. Often, the situation was described where milkers identify CM, but herd managers and owners were consulted before making treatment decisions: “They’ll either call or text me when they see a problem cow” (P4). The manager or owner typically assumed the responsibility of maintaining records for treated cows and their respective mastitis history: “I’m sort of the person that decides and has a database of what cows might be chronic, why they might be treated or not treated if they have been treated multiple times” (P3). When multiple persons on a farm were involved in the CM treatment decision making, a discussion typically only took place when cases were more complex. “If there’s a question about it, then usually [co-owner] and myself will talk: ‘Okay this cow’s got mastitis’. We take a look at her: ‘Is she one we want to cull or is she one we want to treat?’ and make that decision then” (P16). Less complex cases that aligned with protocols typically did not require consultation with other decision makers before acting, explained by one farmer as: “Sometimes there is a little bit of a discussion involved but most of the time it is automatically done” (P15).

It became apparent from the interviews that if multiple decision-makers were present on a farm, their perception of cow value often differed, as well as their experience regarding the consequences of treatment decisions. This influenced treatment decision making. In one interview, these differences were highlighted: “Because he [farm owner 1] is not in here doing all the work. Right? Where she [farm owner 2] and I take turns, we have to treat these cows, find these cows and deal with these cows. And she [farm owner 2] is a lot more ‘out you go’ than he [farm owner 1] ever is” (P17). These varying stances underscore the dynamic interplay between various actors involved in treatment decisions.

##### 3.2.2.2 Dynamics with veterinarians

Interviewed farmers described that veterinarians were consulted to discuss treatment protocols as well as evaluate previous CM cases, causative organisms, the general udder health status of the herd, and chronic cases: “While the decision [to treat] lays with us, he’s [the veterinarian] with herd health. We sit down and discuss some things.” (P8). Urgency was provided as a reason not to consult the veterinarian for the treatment of specific cases, as described by one farmer: “If you wait ‘til tomorrow ‘til the vet comes out, you have waited too long” (P13). Nonetheless, farmers underlined that their herd veterinarians were approachable and easy to reach: “If I have any questions, I’ll reach out to him, and he’ll respond within a couple of hours usually” (P4).

##### 3.2.2.3 Dynamics with milkers

Farmers expressed challenges in ensuring employee adherence to protocols, especially when it came to monitoring cows with few clinical mastitis signs. Another challenge raised was that employees may not always feel entirely comfortable administering antimicrobial treatment: “It depends on who’s milking. If they are, if they know how to treat the cow or not” (P1). Employees sometimes also lack the knowledge to safely administer intramammary antimicrobials or to identify CM cases: “For the past few years, the staff I have aren’t capable. They’re very good milkers, but they just do not have that diagnostic experience and treatment” (P4). Issues with milkers were also encountered when choosing the appropriate treatment: “Although the milkers tend to want to treat [severe cases with] intramammary anyway, the treatment is generally Metacam [meloxicam]” (P3).

#### 3.2.3 Moving beyond protocols

Throughout the interviews, farmers referred to on-farm protocols, which were often oral in nature although they remain a requirement for the mandatory Canadian national quality assurance program proAction. However, when delving deeper and probing into exceptions and exceptional circumstances where deviation from the protocol occurred, the more intricate decision-making process detailed in this manuscript emerged. For example, one interviewed farmer described their protocol with a one-liner: “Treating? Anything over a million” (P8); which was later revised, and a more nuanced picture was given that followed the various considerations depicted in Figure 1. Similarly, for another farmer, when asked if there ever were situations to not treat cows, information about pregnancy and cull list emerged which was not included in their protocol: “If the cow’s a cull, she’s always going to be culled. And if she is only making 20 or 25 kg, we probably would not put the time or effort into that [to start treatment]” (P9). These examples illustrate that ‘exceptional’ situations occur often, and that, in practice, moving beyond the set protocol is more common than is often recognized.

## 4 Discussion

In this study, we examined CM treatment decision making on 15 Western Canadian dairy farms by describing the intricacy of identification and classification of CM, factors considered during antimicrobial treatment decision making as well as subsequent drug choices and considerations around treatment termination. Three overarching themes that influenced this decision-making process were generated: ‘Personal attributes’, ‘Inter-actor dynamics’, and ‘Moving beyond the protocol’.

The presented decision-making diagram aligns with conclusions drawn by Vaarst et al. (15), which covers decisions made based on clinical signs, individual cows, and the entire herd. Nonetheless, our study adds a new layer of information by specifically highlighting decision-making around treatment choice, which includes considerations of alternatives to antimicrobials and urgency of action, an aspect not studied by Vaarst et al. (15). Regardless, the overall similarity between the 2 studies underscores a consistency across 2 decades in Canada and Norway, where farmers are shown to consider the same aspects. Key aspects of the CM decision-making process as indicated in the current study have also been reported in other studies, such as the importance of case severity, SCC, suspected pathogen, and the comparison with other cases on the farm (31, 32). These factors aid the farmer in minimizing uncertainty regarding treatment outcome, an important determinant driving on-farm antimicrobial use (17). Recent work also suggests a variability among farmers in terms of prioritization of these different decision factors (8). However, because the current study approach did not intend to quantify the relative importance of each decision factor, but rather to provide insight into the intricacies and complexities of the decision-making process, no claims can be made regarding variation in the relative importance of each of the factors among our participants.

Our study also describes the process of identifying and classifying CM, highlighting differences between farms with AMS and traditional milking parlors in prioritizing the use of various types of data. Although farms with both types of milking system used milk production and quality data in addition to visual evaluation of the udder to identify CM, AMS farmers generally had access to data from more sensors and more frequent data reports, a benefit also highlighted by Swedish (33) and Ontario farmers (16). These data allow for identifying inflammation without visual evaluation, which is often the starting point of the CM identification process on parlor farms. All AMS farmers in the study reported performing a visual assessment of the udder after evaluation of data reports suggested CM, which has also been reported by German farmers (34). It is important to note, however, that farms with AMS need to regularly review available data to prevent high false-positive rates and to minimize workload (35). This is especially important when built-in algorithms are used to predict the presence of CM, as sensitivity ranges around 60–80% (36). Differences in antibiotic treatment choices between parlor and AMS farms were not assessed in the current study, although research from the Netherlands suggests that AMS farms are more likely to treat with systemic antimicrobials compared to intramammary antimicrobials due to difficulties administering intramammary antimicrobials outside the milking parlor (37).

Other tools used by farmers to identify and characterize CM cases in this study included assessment of rumination and activity data. Decreased rumination is an established indicator of reduced feed intake, which has been associated with the presence of CM (38). Reduced lying time has also been described as one of the behaviors occurring during mastitis (39, 40). Some farmers also employed additional diagnostics in the form of culturing CM milk samples. Most commonly, cultures were employed to identify intramammary infections among fresh cows, although some farmers cultured all CM cases, similar to what has been previously reported by U.S. producers (31). As such, most farmers initiated treatment without awareness of causative agent. Since certain non-severe cases (e.g., those caused by *E. coli* or CM cases where bacteria are not present in the udder) do not require antimicrobial treatment, culturing of confirmed CM cases is recommended before initiating antimicrobial treatment (12).

Non-severe CM cases that were selected for antimicrobial treatment often received only intramammary antimicrobials, with severe cases also receiving systemic antimicrobials, reflecting protocols suggested by research in the United States (41). Although protocols were established with herd veterinarians, veterinarians were rarely consulted for individual cases throughout the decision-making process. Administration of systemic antimicrobials is considered extra label in many countries, and their effectiveness for treating severe CM cases is debatable (12). However, although administration of systemic antimicrobials should only occur after veterinary approval (and preferably after diagnostic testing), the interviews indicated that this was not always the case. Although co-administering NSAIDs has been shown to improve bacteriological and clinical cure outcomes (42, 43), few farmers reported using pain medication for non-severe CM cases, in contrast to frequent use by Danish farmers (32).

Extending CM treatments beyond label prescription was common practice, similar to what has been documented in Germany and the Netherlands (44, 45). The predominant reason for extended treatment in our study was a continuation of clinical signs, which raises concerns about farmers inability to differentiate between intramammary infections versus just the presence of clinical signs. After a normal course of antimicrobial treatment, underlying infections are unlikely to persist for most cases with a bacteriological cause, although signs of inflammation might still be present (46). Although an assessment of a milk sample after treatment is the only way to confirm bacteriological clearance (47), this was not employed by any of the farmers in our study, likely due to time and financial constraints, although this was not covered in the interview. Regardless, awareness of the distinction between infection and inflammation among farmers is essential to prevent unnecessary antimicrobial use and risk of antimicrobial resistance (48).

Through the Canadian dairy industry’s national quality assurance program proAction, dairy farms are required to have standard operating protocols (SOPs) for common antimicrobial treatments. Although such SOPs are valued by farmers (31, 49), farmers are also known to deviate from these protocols (50), similar to results described in this study. Delving deeper into the theme ‘Beyond the protocol’ allows us to make inferences regarding the ‘thinking systems’ used by interviewed farmers. According to dual process theories, cognitive operations can be categorized into 2 thinking systems: intuitive (‘System 1’) and reflective (‘System 2’) (51). The intuitive approach involves quick, automatic decisions, often relying on associations. In contrast, the reflective approach is more deliberate, carefully considering all alternatives before arriving at a decision. The quickness with which farmers summarized their protocols early in the interviews suggests they mainly used an intuitive cognitive system when handling CM cases that fit normal descriptions. However, in complex situations such as CM decision making, cognitive operations can move from reflective to intuitive, as skills and experience develop, in line with observations in our study (52). Only when asked to delve into details of CM treatment decisions, or when discussing cases where treatment expectations were not met, did the controlled, self-aware, reflective approach emerge. This implies that most farmers’ CM decisions are more likely to be automatic and associative, where past experiences and context are considered subconsciously (53).

Use of the intuitive cognitive approach for mild and moderate CM cases can introduce cognitive biases, including choices geared toward short-term gains (54). This may make antimicrobial treatments more appealing than improvements in udder health management. In contrast to the more mentally taxing reflective approach, intuitive cognitive processes place higher value on the first information that comes to mind (the ‘anchoring bias’) and tend to overestimate the likelihood of specific outcomes because they are plausible (54). These heuristic judgments are inherent biases to the intuitive cognitive process and can be mitigated through a deliberate effort to reason (52). However, they represent a challenge when trying to improve CM decision making on the farmer level. Veterinarians, in contrast to farmers, have received extensive training on the use of antimicrobials and their impacts, shaping their intuitive decision-making to include long-term gains. We suggest that interventions aimed at improving CM decision making among farmers should target methods of slowing down the decision-making process, to allow for new cognitive associations to be made which will help make more sustainable CM treatment decisions.

We can also extrapolate the interplay between intuitive and reflective thinking approaches to the theme ‘Personal attributes’, especially regarding the use of different types of experiential knowledge in the decision-making process. Often, farmers displayed assessing experiential knowledge when they drew associations between the current case explored in the interview and previous cases - an example of practices guiding intuitive decisions. When treatment outcome expectations were not met, however, farmers seemed to move to the reflective approach, drawing on other experiences (such as efficacy of medication on previous cases of the same cow) to aid in decision making. The role of experiential knowledge in antimicrobial treatment decision making has also been seen in U.K. and U.S. farmers (17, 49). Rees et al. (17) also described experimental knowledge, which relies on the outcome of deliberate testing and trials of different treatments. Experimental knowledge, however, did not emerge from the interviews in this study; this might indicate that, although it plays a role in other on-farm antimicrobial decision-making processes, this type of decision making might not play an important role in CM decision specifically.

Another overarching theme generated was ‘Inter-actor dynamics’. While most CM treatment decisions were made by the herd manager, regular discussions among the operational team, co-owners, milkers, and veterinarians were common. These discussions revolved around operating protocols and herd goals. Participants described that *ad hoc* meetings between 2 or more actors took place to discuss cases requiring special attention. This behavior implies that actors believed that the group would reach better quality decisions together compared to solo, a characteristic of a synergetic group dynamic (55). In case of treatment decisions for CM cases that do not follow normal patterns, this assumption is likely correct, as each actor holds different pieces of information relevant to the decision making: the milker and the herd manager have daily contact with the affected cows, whereas the owner is more informed on the long-term herd goals, and the veterinarian has insight into the disease risk factors and etiology. A certain decision hierarchy, and thereby power imbalance, also seemed to be present, with owners and older generations having more decision power, a feature also recognized by Rees et al. (17) during interviews with U.K. farmers regarding general on-farm antimicrobial use. This decision hierarchy can lead to tensions, and can be a source of stress, an important aspect to recognize and mitigate when proposing the adoption of novel CM treatment protocols (17).

The role of the veterinarian in on-farm decision making is well-established and documented (45). More specifically, dynamics between veterinarians and dairy farmers have been described extensively (e.g., 56, 57). However, the dynamics between the actors in these decision-making teams on dairy farms have not been described, and therefore, cannot be used to contextualize our findings. Studying group dynamics is complex (58), yet is common practice in healthcare settings (59, 60), and studying team dynamics can help identify areas for improved antimicrobial stewardship, [e.g., (61)]. Other actors – other farmers and nutritionists previously mentioned by Swinkels et al. (45) – were not mentioned in our interviews. This could be attributed to their more indirect impact on treatment decisions, primarily influencing protocols rather than specific case decisions.

The lack of involvement of the veterinarian in case-by-case decision-making can be considered a consequence of the structure of current dairy practice in the region. As part of the shift toward progressive dairy medicine, emphasis has been placed on training producers and on-farm employees to recognize clinical signs of commonly occurring medical conditions, such as CM, and to follow an established protocol. Additionally, feasibility concerns have been described by producers and shed light on the lack of involvement of the veterinarian at this level (62). These include: the availability of the veterinarian to visit the farm for each CM case, the additional cost to the producer, and the geographic landscape of the region (i.e., farms are considered relatively far apart). While the expectation that the veterinarian participate in decision-making regarding each CM case may not be considered practical for producers and veterinarians in this context, direct involvement of the veterinarian in protocol development is important and was not thoroughly discussed by participants in the sample. The data collected through the interview process do not allow us to know that the veterinarian was not involved in the protocol development process. However, they do highlight the lack of involvement of the veterinarian in individual cases and demonstrate that the producer does not view the veterinarian as a first line resource for decision-making regarding CM cases.

The current legal framework in Canada (63) states that a prescription is needed to purchase antimicrobials, and antimicrobials are only to be prescribed within a current veterinarian-client-patient relationship (64). This relationship demands veterinarians having sufficient knowledge on which to base the assessment, diagnosis, and treatment of cases, and clients assuming responsibility to communicate the needs of the animal to the veterinarian. Therefore, based on the current legal framework, the veterinarian should be contacted in cases that do not allow the producer or on-farm employee to easily follow the protocol so that an individual assessment can be made. Based on information gleaned through this study, further assessment of veterinarian-producer relationships is warranted and may allow us to further unpack the lack of involvement of the veterinarian in decision-making surrounding CM.

In reflecting on the lead researchers’ perspectives and position, and its potential impact on the presented results, it is important to note that EdJ’s background in clinical mastitis was primarily theoretical, derived from scientific literature, rather than practical experience—as she is not a veterinarian. This lack of direct clinical experience facilitated an open and non-judgmental environment during the interviews, encouraging farmers to share their practices more freely. Regarding the information power of the final sample size, EdJ’s initial unfamiliarity with qualitative research required additional interviews to cover all topics; as the participating farmers were all involved in their CM treatment decisions and farmed on conventional farms, the narrow study aim was more easily addressed. The differences between farms with AMS and conventional milking systems did require additional interviews to sufficiently describe each situation’s intricacies.” The iterative fashion in which the results were generated by EdJ, including discussions with the rest of the research team, allowed for continuous reflection on the achievement of sufficient information power to answer the research question. In reflecting on the interview method used, the data obtained from Zoom interviews matched the quality and richness of in-person interviews; similar themes and concepts were brought forward. This similarity is consistent with research comparing the quality of Zoom with in-person interviews (65).

While our study has provided valuable insight into CM decision making, it is important to acknowledge several limitations that might impact the findings. Interviews rely on respondents’ ability to accurately recall details about their decision-making process, which can be challenging, considering most of the CM events discussed occurred in the past – sometimes multiple weeks or months ago. An ethnographic approach, where participant and direct observation are at the core of data collection, can help mitigate this issue and provide deeper insights into social dynamics. However, it is important to note that this method is time-consuming and resource intensive. In contrast to focus groups, interviews allow for more in-depth exploration of individual experiences. Although both interviews and focus groups may be susceptible to socially desirable responses from participants, the interviewer took specific measures to build rapport with farmers and maintain a nonjudgmental and curious stance throughout the interviews. Surveys could be employed in future research as they are a tool to capture quantifiable data and allow for describing trends among farmers with AMS and those milking with parlors. Similarly, surveys would allow for inferences regarding the use frequency of described practices and could quantify the reported treatment considerations among the broader dairy farmer population in Canada.

In conclusion, CM treatment decision-making is a multifaceted process, and can be classified into 4 steps: identification and classification, treatment approach, treatment choice, and treatment termination. At each of these steps, farmers considered many different factors leading to CM treatment decisions, including evaluation of milk production and quality data, visual characteristics, systemic signs, additional diagnostics, likelihood of cure, value of the cow, herd goals, urgency of treatment, available drugs, timing of the case, definition of success and progression over time. Three themes shaped these decisions: ‘Personal attributes’, including approach and experiential knowledge; ‘Inter-actor dynamics’, such as shared decision making and dynamics among producers, veterinarians, and milkers; and ‘Moving beyond protocols’, which highlights the dynamic nature of their decision making. While tools and techniques are available to assist farmers in improving udder health and refining antimicrobial choices, understanding the generated themes is key for designing effective interventions for CM that align with the reality of farming operations.

## Data availability statement

The raw data supporting the conclusions of this article will be made available by the authors, without undue reservation.

## Ethics statement

The studies involving humans were approved by the University of Calgary Conjoint Faculties Research Ethics Board (#REB21-0699). The studies were conducted in accordance with the local legislation and institutional requirements. The participants provided their written informed consent to participate in this study.

## Author contributions

EdJ: Conceptualization, Data curation, Formal analysis, Methodology, Writing – original draft, Writing – review & editing. IvdV: Data curation, Formal analysis, Methodology, Writing – original draft. A-MS: Conceptualization, Formal analysis, Methodology, Writing – review & editing. JI: Conceptualization, Formal analysis, Methodology, Writing – review & editing. KR: Conceptualization, Formal analysis, Methodology, Writing – review & editing. DK: Formal analysis, Writing – review & editing. HB: Conceptualization, Methodology, Supervision, Writing – review & editing.
